# Effect of Occupational Exposure to Low-frequency Electromagnetic Fields on Cataract Development

**DOI:** 10.18502/jovr.v20.12281

**Published:** 2025-04-25

**Authors:** Mohammad Hosein Validad, Monireh Mahjoob, Masoud Pishjo, Mostafa Diani, Tahereh Rakhshandadi

**Affiliations:** ^1^Department of Ophthalmology, Alzahra Eye Hospital, Zahedan University of Medical Sciences, Zahedan, Iran; ^2^Health Promotion Research Center/Department of Optometry, Rehabilitation Faculty, Zahedan University of Medical Sciences, Zahedan, Iran; ^3^Refractive Eye Research Center, Mashhad University of Medical Sciences, Mashhad, Iran

**Keywords:** Cataract, Low-frequency Electromagnetic Field, Occupational Exposure

## Abstract

**Purpose:**

Cataracts are the second leading cause of visual impairment worldwide. This study aimed to examine the impact of occupational exposure to low-frequency electromagnetic fields on cataract development.

**Methods:**

One hundred employees of Zahedan Electricity Company participated in this study. They were assigned to four groups based on their level of exposure: regular, operational, operator personnel, and non-exposure. Based on LOCS III grading, the risk of developing different types of cataracts (i.e., nuclear, posterior subcapsular, and cortical) was evaluated for all participants.

**Results:**

The frequency of cataracts was 62.2% in the exposure group (which includes three subgroups: the regular, operational, and operator personnel) and 53.8% in the non-exposure group. There was a significant difference between the study groups in terms of nuclear opacity grading (P = 0.003). The correlation between nuclear and posterior subcapsular cataract grading and work experience duration in the exposure group was statistically significant (P < 0.018).

**Conclusion:**

This study's findings indicate that exposure to low-frequency electromagnetic fields such as power lines, power plants, and power distribution posts may be a risk factor for cataract development, particularly nuclear cataracts.

##  INTRODUCTION

High-voltage electricity transmission lines are pervasive in developed countries and urban areas of many developing nations. The collective term for the electric and magnetic fields produced by power lines is electromagnetic fields (EMFs). The strength of electric and magnetic fields is dependent on the intensity of the current flowing through a conductor and the distance from the source.^[1]^ EMFs produce thermal and nonthermal effects^[2,3]^ that can have detrimental effects at the cellular and molecular levels and can result in functional and structural changes in organ systems. Thermal effects are characterized by a rise in body temperature.^[3]^ The effects of exposure to EMF on human health have been reported in previous studies.^[4]^ Examples include the impact of this exposure on leukemia,^[5,6]^ brain and breast cancer,^[7]^ neurodegenerative diseases,^[8]^ Alzheimer's disease,^[9,10]^ and depression^[8,11]^ as a result of an alteration in the daily rhythm of pineal melatonin production and excretion.^[12]^

The eye is a water-rich organ with a high EMF power absorption capacity and few blood vessels, and it is less likely to disperse thermal energy via perfusion.^[13,14]^ Due to their low blood supply, the eyes are among the most thermally vulnerable areas of the body. Exposure to high-power-density microwaves can adversely affect the eyes and provoke significant biological alterations via thermal mechanisms.^[13,15]^

Accounting for roughly 20 million cases, cataracts are the leading cause of blindness and the second most common cause of visual impairment globally.^[16]^ Age plays a significant role in the development of cataracts, while heredity is the primary cause of the condition.^[17]^ Other risk factors, such as systemic diseases, smoking, and excessive sunlight exposure, can also cause cataracts.^[17]^ Besides, research findings indicate that cataracts can be triggered by thermal and nonthermal effects of microwave-electromagnetic radiation.^[15,18]^

The number of workers who are occupationally exposed to EMFs is exceptionally high. Indeed, the vast majority of the worker community may be considered exposed, suggesting a potential occupational health risk. There exist few studies on cataract development in workers exposed to EMFs. Therefore, this study aimed to examine the incidence of cataracts in this population at Zahedan Electricity Company.

##  METHODS

One hundred employees working at Zahedan Electricity Company who were regularly exposed to EMFs in the company's power plants, power lines, and distribution posts were recruited in this cross-sectional study. This study was derived from an ophthalmology residency thesis approved by the Ethics Committee of Zahedan University of Medical Sciences (ethical code: IR.ZAUMS.REC.1397.045) and followed the Declaration of Helsinki. All participants signed an informed consent form.

The recruited employees were assigned to one of four groups based on the duration of exposure:

1. Operators (who were exposed to 50 HZ electromagnetic waves for over 170 hours a month);

2. Operational staff (employees exposed to 50 HZ electromagnetic waves for 70 to 170 hours a month);

3. Regular staff (those exposed to 50 HZ electromagnetic waves for less than 70 hours each month); and

4. The non-exposure group (electric company employees who were not exposed to EMFs).

All participants completed a general health questionnaire and underwent a comprehensive ophthalmic examination, which included complete patient history, ophthalmoscopy, biomicroscopy, and noncontact tonometry (Topcon CT-1/CT-1P, Tokyo, Japan). Best-corrected visual acuity of all participants was measured by the Snellen chart at 6 meters. Eye diseases such as strabismus, glaucoma, and amblyopia, and a history of trauma or eye surgery were the exclusion criteria. All participants had at least one year of continuous work experience.

All participants were examined by two ophthalmologists using a slit lamp (Topcon SL-D7) and the Lens Opacities Classification System III (LOCS III) to determine the presence and grading of cataracts.^[19]^ The eye with the greater opacity was chosen in individuals whose opacity varied in both eyes. The ophthalmologists who evaluated the cataracts were unaware of the study groups.

SPSS version 21 (IBM, Chicago, USA) was utilized for statistical analysis. Kolmogorov–Smirnov test showed that quantitative variables had a normal distribution (P > 0.065). One-way ANOVA was used to determine whether there were any significant differences in age and work experience duration among the study groups. We performed a Kruskal–Wallis test to determine the frequency of opacities grading within study groups. Spearman's correlation was used to evaluate the relationship between years of work experience (quantitative variable) and crystalline lens opacity grading (qualitative variable). Moreover, binary logistic regression analysis was used to examine the odds ratio of cataracts, and the independent variables used for this analysis included age, work experience, and study groups. P-values < 0.05 were regarded as statistically significant.

##  RESULTS

One hundred male employees of Zahedan Electricity Company participated in the study. Participants were categorized as employees without exposure (non-exposure group; n = 26) and exposure groups, including regular personnel (n = 19), operation personnel (n = 22), and operators (n = 33). The mean and standard deviation of age and work experience for all participants are displayed in Table 1. There was no significant difference in age (P = 0.543) or work experience duration (P = 0.713) between the study groups.

In the exposure group, the frequency of cataracts was 62.2% (n = 46) – regular personnel: 52.6% (n = 10), operational personnel: 45.5% (n = 10), and operators: 78.8% (n = 26) – while in the non-exposure group, the frequency was 53.8% (n = 14). The adjusted odds ratio for cataracts in the study groups was 1.40 (95% CI: 0.57–3.47).

The frequencies of opacity grading for nuclear, posterior subcapsular (PSC), and cortical opacity in the exposure and non-exposure groups are displayed in Table 2. The grading of nuclear opacity varied significantly between these groups (P = 0.003); specifically, this difference was significant between the non-exposure group and operators (P = 0.003). Other opacity gradings did not differ significantly between the study groups (P = 0.260 for PSC and P = 0.562 for cortical opacity).

Visual acuity in the exposure and non-exposure groups was 0.92 ± 0.09 and 0.96 ± 0.06 decimal, respectively, which indicates a significant difference (P = 0.021).

Table 3 depicts Spearman's correlation between years of work experience and crystalline lens opacity grading. Significant correlations were found between nuclear and PSC cataract grading and work experience in the exposure group (P < 0.001, r = 0.542 for nuclear cataracts and P = 0.018, r = 0.274 for PSC cataracts).

The adjusted odds ratio for work experience and cataract was 1.25 (95%CI: 1.03–1.51; P = 0.018). The effect of age on cataract development was not statistically significant (P = 0.31).

**Table 1:** The mean ± SD and 95% CI of age and work experience of the study groups.

	**Non-exposure group**	**Exposure group**				P-value*
		**Regular personnel**	**Operational personnel**	**Operator**	**Total (%)**	
No.	26	19	22	33	74	
Age	37.07 ± 9.04 (33.42, 40.72)	39.94 ± 10.01 (35.12, 44.76)	40.04 ± 7.49 (36.72, 43.37)	40.15 ± 9.10 (36.92, 43.37)	40.06 ± 8.78 (38.08, 42.09)	0.543
Work experience	12.92 ± 7.70 (9.80, 16.03)	13.31 ± 8.24 (9.34, 17.29)	14.68 ± 5.93 (12.04, 17.31)	14.75 ± 5.43 (12.47, 17.03)	14.36 ± 6.74 (12.85, 15.89)	0.713
${\;}^{*}$ *P*-value for one-way ANOVA						

**Table 2:** The frequency of cataract grading for nuclear, posterior subcapsular, and cortical opacity.

**LOCS III grade**		**Non-exposure group**	**Exposure group**				***P*** -value ${\;}^{*}$
			**Regular personnel**	**Operational personnel**	**Operator**	**Total (%)**	
Nuclear	Normal	14 (42%)	9 (47%)	12 (54%)	8 (24%)	29 (29%)	0.003
	NO1	11 (54%)	4 (21%)	5 (23%)	8 (24%)	17 (17%)	
	NO2	1 (4%)	4 (21%)	4 (18%)	13 (39%)	21 (21%)	
	NO3	0	0	1 (5%)	4 (13%)	5 (5%)	
	NO4	0	2 (11%)	0	0	2 (2%)	
Posterior subcapsular	Normal	24 (92%)	17 (90%)	21 (96%)	25 (76%)	63	0.260
	P1	0	1 (5%)	0	4 (12%)	5 (5%)	
	P2	2 (8%)	1 (5%)	1 (4%)	4 (12%)	6 (6%)	
Cortical	Normal	26 (100%)	19 (100%)	23 (100%)	32 (97%)	73 (73%)	0.562
	C1	0	0	0	1 (3%)	1 (1%)	
${\;}^{*}$ *P*-value for Kruskal–Wallis analysis							

**Table 3:** Spearman's correlation between work experience and crystalline lens opacity in the study groups.

		**Non-exposure group**	**Exposure group**			
			**Regular personnel**	**Operational personnel**	**Operator**	**Total**
Nuclear cataract grading	*P*-value ${\;}^{*}$	0.608	0.007	0.023	0.001	< 0.001
	Correlation coefficient (*r*)	0.106	0.599	0.432	0.570	0.542
Posterior subcapsular cataract grading	*P*-value	0.216	0.063	0.097	0.267	0.018
	Correlation coefficient (*r*)	0.251	0.433	0.363	0.199	0.274
Cortical opacity cataract grading	*P*-value	*N* = 0	*N* = 0	*N* = 0	0.232	0.243
	Correlation coefficient (*r*)	*N* = 0	*N* = 0	*N* = 0	0.214	0.137
${\;}^{*}$ *P*-value for Spearman's correlation analysis						

##  DISCUSSION

There are two primary types of EMFs: extremely low frequency (ELF) waves and radiofrequency (RF) waves. ELFs can be produced by electrical lines or transmission towers, which have been the subject of research for decades. Eyes are susceptible to high EMF absorption due to the lack of extensive blood vessels in this organ.^[14]^ This study found that the risk factor of EMF exposure for crystalline lens opacity was 1.40% higher in the group exposed to electromagnetic waves than in the non-exposure group.

Increased eye temperature due to EMF has been evaluated computationally in a variety of scenarios, including plane wave exposure,^[20,21]^ blood perfusion,^[14]^ specific absorption rate variability due to head shape,^[22]^ and dielectric properties of the eye.^[14,23]^ According to the findings of the present study, crystalline lens opacity, particularly nuclear cataracts, was significantly higher among participants who were exposed to EMF at work, primarily among operators with longer periods of daily exposure [Table 2]. Per our findings, long-term daily exposure to EMF was associated with significantly more adverse health effects compared with short-term daily exposure.^[24]^ Although there is limited data regarding the effects of EMF exposure on the eye and visual function, animal studies indicate that microwave-induced cataracts are caused by increased body temperature.^[15,25]^ This idea is the focus of several studies that have sought to investigate the link between rising temperatures and the onset of cataracts.^[15,26]^ Furthermore, confirming biochemical changes in lenses exposed to RF energy.^[27]^ Ascorbic acid levels have been found to decrease in lenses exposed to microwave power. The ascorbic acid concentration in the lens drops as the microwave power increases.^[27]^ It is reasonable to assume that these biochemical alterations result from thermal effects, given that cataractogenic exposure levels increase lens temperature. Human populations exposed to RF energy have shown ocular effects in some studies, however, other studies have not replicated these findings.^[26,28,29]^ This discrepancy may be partially explained by differences in EMF exposure levels or patterns between studies. Moreover, given the few studies in this domain, more extensive research is required to shed light on the relationship between EFM exposure and the risk of crystalline lens opacity, among individuals with long-term daily exposure.

Our findings revealed a significant correlation between the exposure group's length of work experience and nuclear opacity grading. Although the correlation between longer employment and EMF side effects has been reported in previous studies, the number of these studies is limited.^[24,30]^

The present study had several limitations. One of these limitations was the lack of imaging techniques to assess lens density. However, according to previous studies, these techniques correlate well with LOCS III,^[31,32]^ which confirms the results of our study. Another limitation of this study was the small sample size. Although the sample size of this study was larger than in previous studies,^[18,22,28]^ it is recommended to conduct further research with a larger sample size and with other lens opacity assessment tools.

In summary, according to the findings of this study, occupational exposure to EMFs is linked to a higher risk of cataract formation, particularly nuclear cataracts. Consideration should be given to the occupational effects of EMF exposure among people working for electricity companies.

##  Financial Support and Sponsorship

None.

##  Conflicts of Interest

None.
